# Robotic Surgery in Pelvic Organ Prolapse: A Retrospective Comparison of Ileopectopexy and Sacrocolpopexy

**DOI:** 10.7759/cureus.72356

**Published:** 2024-10-25

**Authors:** Deerush Kannan, Amrithavarshini Ragavan, Madhav Tiwari, Narasimhan Ragavan, Meera Ragavan

**Affiliations:** 1 Urology, Apollo Hospitals, Chennai, IND; 2 Urogynaecology, Apollo Hospitals, Chennai, IND

**Keywords:** female urology and uro oncology, minimally invasive surgical procedures, pelvic organ prolapse, robotic surgical procedures, robotic urology

## Abstract

Introduction: Pelvic organ prolapse (POP) is a common condition affecting over half of women above the age of 50. Sacrocolpopexy is a widely recognized surgical approach for apical prolapse, restoring the vaginal axis effectively. Despite its efficacy, complications such as bowel dysfunction and stress urinary incontinence (SUI) are common. Ileopectopexy, a newer technique utilizing the iliopectineal ligament, offers an alternative that may reduce complications while maintaining efficacy. This study compares the outcomes of robotic sacrocolpopexy and ileopectopexy in a tertiary care setting.

Materials and methods: This study retrospectively analyzed the collected database of 35 patients who underwent robotic sacrocolpopexy or ileopectopexy between 2021 and 2023. Surgeries were performed using either the Da Vinci or Hugo robotic systems. Parameters studied included console time, blood loss, complications, hospital stay, recurrence rates, and follow-up duration. Data was statistically analyzed using SPSS (IBM Corp., Armonk, NY, v28.0), with a p-value of <0.05 considered significant.

Results: Both groups had similar age distributions and presenting symptoms, most commonly complete uterine prolapse. Ileopectopexy had no complications, while sacrocolpopexy saw two vaginal lacerations. Blood loss was minimal across all cases (<100 mL). Post-operative bowel complaints were more frequent in the sacrocolpopexy group (14%), but absent in ileopectopexy. Hospital stay, console time, and additional procedures were comparable. Follow-up was longer for sacrocolpopexy (34.8 vs. 5.3 months).

Conclusion: Both robotic sacrocolpopexy and ileopectopexy effectively managed apical prolapse with minimal blood loss and comparable hospital stays. However, ileopectopexy showed fewer bowel complications, making it a promising alternative. More extensive studies with longer follow-ups are needed to validate these findings.

## Introduction

Pelvic organ prolapse (POP) affects millions of women, with more than half of the female population over 50 years [1]. Apical prolapse, the downward displacement of the vaginal apex, uterus, or cervix, manifests in symptoms such as vaginal bulging, pelvic pain, dyspareunia, and urinary and bowel dysfunctions [2,3].

Sacrocolpopexy is the most effective surgical solution for apical prolapse, with sacrocolpopexy being particularly adept at restoring the vagina's physiological axis. Unlike traditional abdominal sacrocolpopexy, minimally invasive techniques avoid large incisions and extensive bowel manipulation, resulting in quicker recovery [4]. Despite its effectiveness, sacrocolpopexy can cause complications, including defecation disorders and stress urinary incontinence (SUI) [5]. Gastrointestinal issues like small bowel obstruction and ileus are reported in 0.1% to 5% of cases, often due to reduced pelvic space, adhesions, or nerve trauma [6]. The most serious intraoperative complication is presacral haemorrhage, which can be life-threatening [7-9].

POP is more prevalent among obese patients, who particularly benefit from laparoscopic or robotic surgery due to its minimally invasive nature [9]. However, surgical challenges can arise from the restricted field [10]. In 2007, Banerjee and Noe introduced a new endoscopic technique specifically for obese patients, utilizing the iliopectineal ligament for mesh fixation [11]. This approach avoids narrowing the pelvic outlet and keeps the hypogastric vessels at a safe distance. The iliopectineal ligament, an extension of the lacunar ligament along the pectineal line of the pubic bone, is stronger than the sacrospinous ligament and the arcus tendineus [12]. This robust ligament holds sutures well and provides ample material for pelvic floor reconstruction. It is located at the second sacral vertebra (S2), which is optimal for maintaining the vagina's physiological axis [12].

In this study, we analyzed and presented the data based on a comparison of outcomes between sacrocolpopexy and iliopectopexy as we transitioned from sacrocolpopexy to ileopectopexy in view of the favourable outcomes reported in the literature in the last decade.

## Materials and methods

Study design

This retrospective study utilized a database collected during the years 2021-2023. We included patients in a nonrandomized manner, with the initial 21 patients in the series undergoing sacrocolpopexy, and the subsequent 14 patients undergoing ileopectopexy, as the surgeon transitioned from the former to the latter for POP treatment.

Study population

Patients who presented with POP to the department of urogynaecology undergoing robotic assisted surgical management formed the study population.

Inclusion criteria

All patients undergoing robotic-assisted POP repair, either by sacrocolpopexy or ileopectopexy, during the study period were included.

Exclusion criteria

We excluded any patients who underwent open surgical procedures or had recurrent POP. Other exclusion criteria were patients who did not return for initial follow-up and who were not willing to consent to the study.

Ethics approval

The study was approved by the Institutional Ethics Committee, Biomedical Research (AMH C-S-034- 5/24). Prior consent was obtained from all the patients involved in the study.

All surgical procedures were performed by the same surgeon using either the da Vinci or Hugo robotic systems. The parameters studied included the type of prolapse, type of surgery, console time, dock time, blood loss, perioperative complications, hospital stay, and recurrence rates based on clinical examination up to a six-month follow-up. The data of the patients who are on follow-up beyond the six-month period are also included.

Surgical differences

The standard steps of laparoscopic ileopectopexy and sacrocolpopexy were followed; however, only the patients who underwent sacrocolpopexy were offered a mesh.

Statistical analysis

Descriptive statistics were presented with frequency (percentage). Chi-square/Fisher’s exact test determined the association between two independent categorical factors. A P-value < 0.05 is considered statistical significance. All the statistical analysis was performed using SPSS (IBM Corp., Armonk, NY, 28.0).

## Results

In our study population, the age distribution, presenting symptoms, and associated conditions were similar between the ileopectopexy and sacrocolpopexy groups. The most common presentation in both groups was complete uterine prolapse (n=9 in the ileopectopexy group; n=18 in the sacrocolpopexy group), with comparable rates of associated cystocele and rectocele (Table 1).

**Table 1:** Demographic factors LUTS: lower urinary tract symptoms; SUI: stress urinary incontinence; BOO: bladder outlet obstruction

Parameters	Group, n (%)		Total (n=35)	p-value
	Ileopectopexy (n=14)	Sacrocolpopexy (n=21)		
Age in years				
30–40	0 (0)	3 (14.3)	3 (8.6)	0.475
51–60	2 (14.3)	3 (14.3)	5 (14.3)	
61–70	6 (42.9)	9 (42.9)	15 (42.9)	
>70	6 (42.9)	6 (28.6)	12 (34.3)	
Symptoms				
Mass descending per vagina	14 (100)	21 (100)	35 (100)	>0.99
Associated symptoms				
No	11 (78.6)	16 (76.2)	27 (77.1)	0.098
Abdominal pain	0 (0)	1 (4.8)	1 (2.9)	
LUTS	0 (0)	4 (19)	4 (11.4)	
Post-menopause bleed	1 (7.1)	0 (0)	1 (2.9)	
SUI	2 (14.3)	0 (0)	2 (5.7)	
Comorbidities				
Present	7 (50)	14 (66.7)	21 (60)	0.483
Absent	7 (50)	7 (33.3)	14 (40)	
Diagnosis				
Complete vault prolapse	9 (64.2)	18 (85.7)	27 (77.1)	0.194
Uterine prolapse	3 (21.4)	1 (4.8)	4 (11.4)	
Uterine descent	1 (7.1)	0 (0)	1 (2.9)	
Uterocervical descent	0 (0)	1 (4.8)	1 (2.9)	
Uterovaginal prolapse	0 (0)	1 (4.8)	1 (2.9)	
Procidentia	1 (7.1)	0 (0)	1 (2.9)	
Additional diagnosis (n=11)				
Cystocele	3 (50)	2 (40)	5 (45.5)	0.291
Rectocele	1 (16.7)	0 (0)	1 (9.1)	
SUI	1 (16.7)	0 (0)	1 (9.1)	
BOO	0 (0)	2 (40)	2 (18.2)	
Left ovarian cyst	0 (0)	1 (20)	1 (9.1)	
SUI + Cystocele	1 (16.7)	0 (0)	1 (9.1)	

The length of hospital stay and the need for additional procedures (e.g., hysterectomy) were comparable between the two groups. Specifically, 43% (n=6) of patients underwent hysterectomy in the ileopectopexy group, while in the sacrocolpopexy group, it was 10% (n=2). However, the type of robotic procedure differed significantly (p<0.05); ileopectopexy was predominantly performed using the Hugo RAS system (n=11), while sacrocolpopexy was performed using the Da Vinci robotic system (n=19) (Table 2).

**Table 2:** Clinical factors BSO: bilateral salphingo oophorectomy; TLH: total laparoscopic-assisted robotic hysterectomy; TVT: trans vaginal tape

Parameters	Group, n (%)		Total (n=35)	P-value
	Ileopectopexy (n=14)	Sacrocolpopexy (n=21)		
Hospital stay				
1 day	9 (64.3)	11 (52.3)	20 (57.1)	0.728
>1 day	5 (35.7)	10 (47.6)	15 (42.8)	
Additional procedure				
No	6 (42.9)	11 (55)	17 (50)	0.107
BSO	0 (0)	1 (5)	1 (2.9)	
TLH + BSO	6 (42.9)	2 (10)	8 (22.8)	
Urethral dilatation	0 (0)	4 (20)	4 (11.8)	
Cystoscopy	1 (7.1)	1 (5)	2 (5.9)	
Serosal tear repair	0 (0)	2 (9.5)	2 (5.9)	
Cystoscopy TVT + Bladder suturing	1 (7.1)	0 (0)	1 (2.9)	
Robotic platform used				
Hugo	11 (78.5)	2 (9.5)	13 (37.1)	<0.001
Davinci	3 (21.4)	19 (90.5)	22 (62.8)	
Complication				
Yes	0 (0)	2 (9.5)	2 (5.7)	>0.99
No	14 (100)	19 (90.5)	33 (94.3)	
Blood Loss				
<100 ml	14 (100)	21 (100)	35 (100)	>0.99
Dock time				
<10 mins	11 (78.6)	19 (90.5)	30 (85.7)	0.359
>10 mins	3 (21.4)	2 (9.5)	5 (14.3)	
Console time				
<1 hour	9 (69.2)	13 (61.9)	22 (64.7)	0.727
Bulge				
Yes	2 (14.3)	-	2 (5.7)	0.202
No	12 (85.7)	21 (100)	33 (94.2)	
Bowel complaints/pelvic pain				
Yes	0 (0)	3 (14.2)	3 (8.6)	0.530
No	14 (100)	18 (85.7)	32 (91.4)	
Urinary symptoms				
Yes	3 (21.4)	4 (19)	7 (20)	0.961
No	11 (78.5)	17 (81.0)	18 (80)	

In terms of complications, there was no complication in the ileopectopexy group, while two complications occurred in the sacrocolpopexy group (both patients had vaginal lacerations). Blood loss across all patients was minimal, with all cases reporting less than 100 mL.

Patients in the sacrocolpopexy group had a longer follow-up duration, as many were part of the initial series of cases. The mean follow-up for sacrocolpopexy was longer (34.8 ± 16.6 months), whereas patients undergoing ileopectopexy had a mean follow-up of 5.3 ± 2.7 months.

Post-operative bowel complaints, such as constipation and increased frequency of bowel movements, were noted in 14% of patients in the sacrocolpopexy group (n=3), but were absent in the ileopectopexy group. Urinary symptoms, such as hypercontinence, were not reported in both groups. Two patients in the sacrocolpopexy group were noted to have vaginal lacerations after the removal of the uterus, which were repaired intraoperatively.

## Discussion

Our study shows that the outcomes of ileopectopexy and sacrocolpopexy are comparable in terms of presenting symptoms, associated conditions, length of hospital stay, and the need for additional procedures. Both techniques showed similar efficacy in managing complete uterine prolapse with minimal intraoperative blood loss, reinforcing the safety of robotic-assisted surgery for these procedures.

The safety and efficacy of laparoscopic ileopectopexy have been studied by Kale et al. [13]. In an Indian randomized trial by Khoiwal et al., it was reported that patients who underwent sacrocolpopexy and ileopectopexy had similar outcomes based on quality-of-life (QOL) questionnaires, with the complication rate being the same in both groups [14]. However, in their study, the mesh was placed in all patients, whereas in our study population, only those who underwent sacrocolpopexy were offered mesh.

The difference in the use of robotic systems - Hugo RAS for ileopectopexy and Da Vinci for sacrocolpopexy - did not appear to significantly influence overall clinical outcomes, although the choice of platform may reflect institutional preferences or surgeon familiarity. Gilleran et al. reported their outcomes on robotic sacrocolpopexy with mesh repair. At the same time, our study is unique in comparing the outcomes of two different robotic procedures performed on two different robotic platforms [15]. In contrast, in Gilleran’s study, all patients underwent sacrocolpopexy with the Da Vinci system.

Post-operative constipation and pelvic pain were more frequent in the sacrocolpopexy group. The absence of such issues in the ileopectopexy group could suggest a potentially favourable outcome with respect to bowel function. Forsgren and Altman reported a higher incidence of bowel complaints in patients undergoing sacrocolpopexy after hysterectomy, which is consistent with findings in our group and aligns with most of the reported literature that does not favour sacrocolpopexy [16].

Both groups in our study reported no urinary symptoms post-surgery. In our study, follow-up was notably longer for sacrocolpopexy patients due to their inclusion in an earlier case series, while ileopectopexy patients underwent the procedure after a transition period during which the Hugo Medtronic system was launched, resulting in a relatively shorter follow-up for this group. The shorter follow-up in the ileopectopexy group may have influenced the detection of post-operative complications. Moreover, both groups in the study noted a lack of standardized questionnaires.

While these findings are promising, the limited follow-up duration, particularly in the ileopectopexy group, and the small sample size highlight the need for further longitudinal studies. Additional research with larger cohorts and extended follow-up will be crucial to fully understanding the long-term outcomes and potential benefits of each procedure.

## Conclusions

In conclusion, robotic-assisted ileopectopexy and sacrocolpopexy demonstrated comparable outcomes in managing complete uterine prolapse, with minimal intraoperative blood loss and similar complication rates, regardless of the robotic system used. However, robotic sacrocolpopexy with mesh was associated with a higher incidence of post-operative bowel complications. Further studies with larger sample sizes and extended follow-up are necessary to better define the long-term benefits of each approach.
